# Corruptive Reprograming of Macrophages into Tumor-Associated Macrophages: The Transcriptional, Epigenetic and Metabolic Basis

**DOI:** 10.3390/cancers15174291

**Published:** 2023-08-28

**Authors:** Aamir Ahmad

**Affiliations:** 1 Translational Research Institute, Academic Health System, Hamad Medical Corporation, Doha 3050, Qatar; aamirahmad100@gmail.com; Tel.: +974-44390984; 2 Dermatology Institute, Academic Health System, Hamad Medical Corporation, Doha 3050, Qatar; 3 Department of Dermatology and Venereology, Rumailah Hospital, Hamad Medical Corporation, Doha 3050, Qatar; 4 Department of BioEngineering, Integral University, Lucknow 226026, UP, India

The tumor microenvironment (TME) is an important place with regard to the growth and sustenance of tumor cells. It comprises an area within close proximity of tumor cells and is marked by the presence of several different cell types, such as fibroblasts, extracellular matrix, immune cells, etc., that regularly communicate with the tumor cells [1]. Within a TME are macrophages, which are immune cells that are critical components of the innate immune system [2]. The role of macrophages as antigen-presenting cells (APCs) is important for T-cell activation and function [3], in addition to their roles in fighting bacterial and other infections, clearing cell debris, and tissue repair and remodeling [2,3,4]. Macrophages are referred to as ‘tumor-associated macrophages (TAMs) when they start supporting tumor growth through a positive regulation of angiogenesis, cell proliferation, and even metastasis [5,6]. The article by Larionova et al. [7], entitled ‘Transcriptional, Epigenetic and Metabolic Programming of Tumor-Associated Macrophages’, comprehensively covers the mechanisms that help reprogram macrophages in the TME to become TAMs. The review article discusses such programming at transcriptional, epigenetic, and metabolic levels, which seems to be essential for the unique functional plasticity of TAMs. 

The precursors of macrophages are monocytes, which remain in the bloodstream for a short duration before they migrate into tissues and differentiate into macrophages. In addition, it is worth noting that there are indications for the non-monocyte origin of macrophages [8]. Activated macrophages are two very distinct types, M1 and M2, with the M1 macrophages being the classically activated macrophages that perform the normal functions of macrophages and the M2 being the alternatively activated macrophages, or TAMs, which are known for their immunosuppressive and tumor-supporting abilities [6,9]. The classical M1 macrophages are pro-inflammatory, while the M2 macrophages are anti-inflammatory. However, as rightly pointed out by Larionova et al. [7], this classification of macrophages is not extremely accurate, and macrophages in vivo are quite diverse cells and are often characterized by a certain combination of M1 and M2 traits and functions. 

This article begins with a discussion on the role of several transcription factors that have been documented to play a role in macrophage polarization. Specific transcription factors that are discussed in considerable detail include PU.1, the STAT family, NF-κB, c-Myc, interferon regulator factors (IRFs), the Snail family, and the Maf family. As is well known, transcription factors influence the transcription of genes by binding to target DNA sequences, and, interestingly, more than half of the known transcription factors in the human genome are expressed in macrophages under different states of polarization [10,11]. These transcription factors take cues from signals within the TME and facilitate the reprograming of macrophages with desired inflammation-regulating properties. 

The discussion then moves on to epigenetic changes that help reprogram macrophages, including DNA methylation, histone modifications, and regulation involving miRNAs. Based on the studies on DNA methyltransferases (DNMTs), specifically DNMT1, DNMT3A, and DNMT3B, the authors discuss how these DNMTs have been linked to differential macrophage activation and polarization, particularly under different stimuli. It is concluded that DNA methylation potentially plays a more prominent role in the inflammatory behavior of macrophages compared to the formation of TAM phenotypes, or at least there is a lack of data on the subject. DNA methylation, or the transfer of a methyl group to DNA, is linked to reduced transcription and, therefore, reduced eventual expression of gene products. Histone modifications involve various events, such as methylation, acetylation, phosphorylation, ubiquitination, sumoylation, etc., on the histone proteins that affect chromatin packing [12] and gene expression. Acetylation and methylation are found to be the most frequent histone modifications in macrophages [13], with lysine being the most frequently modified amino acid [14]. Most of the available data on histone modifications in the context of macrophages appear to be directed towards their role in M2 polarization, i.e., the immunosuppressive phenotype [7], although some reports have connected individual histone modification events, such as decreased expression of lysine-specific histone demethylase 1A [15], with the classical M1 polarization of macrophages [7]. Clearly, information on the detailed role of all different histone modifications, particularly in various human cancers, is still emerging. In addition to DNA methylation and histone modifications, the regulation of small non-coding RNAs, the miRNAs, has been investigated in macrophage polarization and function for many years now [6]. This is phenomenal because, even though we are now aware of the great regulatory potential of non-coding RNAs [16,17], not many years ago, these non-coding RNAs were considered ‘junk’ [18]. The article then summarizes some examples of miRNA-mediated effects on macrophages. Interested readers can find detailed information on the topic elsewhere, as this represents one of the areas that has been relatively well studied [6,19,20]. 

Subsequent to the discussion on transcription factors and epigenetic events, this article focuses on the metabolic regulation of macrophage plasticity. Based on the available information, the M1 macrophages seem to be more ‘normoxic’ as compared to the M2 macrophages that reside in the comparatively more inner cores of the TME marked by ‘hypoxia’. As expected, M1 macrophages favor metabolic changes that support increased inflammation, including high glycolytic metabolism through the pentose phosphate pathway, fatty acid synthesis required for inflammatory signaling and impaired mitochondrial oxidative phosphorylation, and the tricarboxylic acid cycle [21]. M2 macrophages, on the other hand, reprogram the metabolism towards oxidative metabolism for bioenergetic purposes, fatty acid oxidation, decreased glycolysis, decreased metabolism via the pentose phosphate pathway, and upregulation of arginase 1 [7]. The article then details the key metabolic features of M1 as well as the M2 polarized macrophages before focusing on the metabolic interactions of TMAs with the tumor cells within TME. The high energy and nutrient demand combined with the competition for oxygen and nutrients among the various components of TME leads to several alterations in the metabolism of TME components. One well-characterized change is the Warburg effect, which is marked by increased glycolysis even under aerobic conditions. In addition to the effects of this changed metabolism in tumor cells leading to the accumulation of lactate and the resulting pro-angiogenic and immunosuppressive phenotype, the article also discusses the available literature indicating TAM-dependent metabolic re-programing of tumor cells to aerobic glycolysis [22,23] and the role of hypoxia [24] and fatty acid oxidation [25] in promoting the tumor-supporting function of TAMs. While the metabolism of macrophages has received considerable attention, it is now apparent that even tumor cells can influence macrophage polarization and maturation through the modulation of their metabolic pathways, thus further highlighting the need to better understand the complex crosstalk between the various TME components. This article makes a good case for the need to further elucidate mechanisms behind macrophages’ polarization so that appropriate therapeutic strategies can be developed to counter the immunosuppressive function of TAMs and restore the efficacy of anticancer medications.
